# Cellular heterogeneity and patterning strategies as revealed by upper respiratory epithelium single cell atlas

**DOI:** 10.1016/j.isci.2025.112845

**Published:** 2025-06-07

**Authors:** Alexander G. Foote, Xin Sun

**Affiliations:** 1 Department of Pediatrics, University of California, San Diego, Cellular and Molecular Medicine, La Jolla, CA 92092, USA; 2 Division of Biological Sciences, University of California, San Diego, San Diego, CA 92093, USA

**Keywords:** Cellular physiology, Organizational aspects of cell biology, Biology of human development, Complex systems

## Abstract

The upper respiratory tract, spanning the pharyngolaryngeal to tracheobronchial regions, enables breathing, vocalization, and frontline defense against airborne insults. We generated a cellular and molecular atlas of the mouse upper respiratory epithelium from pharynx/larynx to tracheobronchial carina by combining single-cell RNA sequencing with spatial validation. Our analysis revealed 18 epithelial cell types, organized into three spatially distinct compartments: *Tmprss11a*+ pharyngolaryngeal, *Nkx2-1*+ tracheobronchial, and *Dmbt1*+ submucosal glands. Stratified squamous pharyngolaryngeal zones displayed extensive and region-specific Keratin codes. Within the pseudostratified tracheobronchial epithelium, diverse luminal cells, including multiple varieties of club cells, exhibit marker-expression gradients along the proximal–distal axes. Lastly, analysis of the submucosal gland epithelium —which contains various cell types, including distinctive myoepithelial cells— revealed extensive diversity both among and within its cellular populations. This spatially resolved transcriptomic atlas elucidates how epithelial identity varies along the upper respiratory axes and will guide investigations into cellular dynamics in health and disease.

## Highlights

• 18 cell types in pharyngolaryngeal, tracheobronchial, and submucosal regions
• Keratin codes and “stress-induced” KRT6A/KRT17 at homeostasis
• Distinct basal and luminal cell characteristics along the proximal-distal axis
• Markers for diverse submucosal gland cells including mucous and myoepithelial cells

## Graphical abstract

**Figure:**
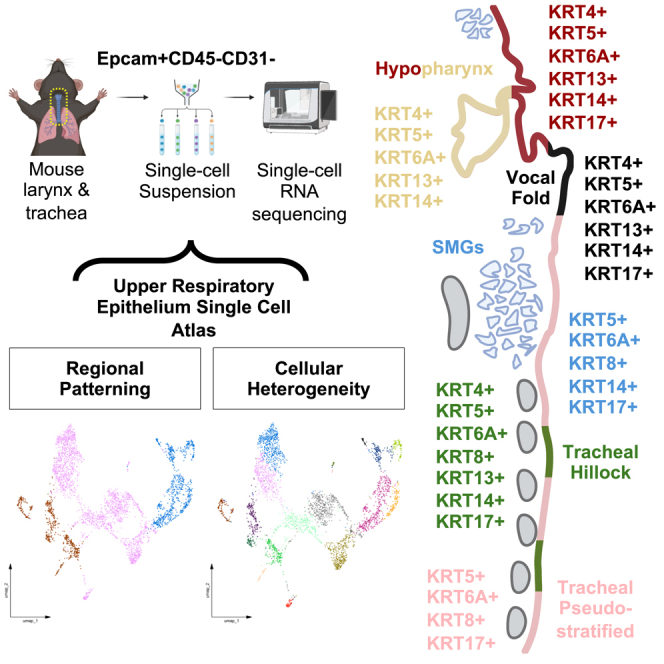

## Introduction

The respiratory tract encompasses the nasal cavity, pharynx/larynx (throat), trachea, bronchi, and lungs, operating as a connected and interdependent system. Mucosal surfaces lined by epithelial cells are essential elements of the respiratory tract, effective not only as a first-line physical barrier against chronic external threats but also for host immune defense, and injury repair.^1^^,^^2^^,^^3^^,^^4^ The epithelium is vital for maintaining respiratory health. Conversely, it is also the site for pathogenesis of an array of upper airway acute and chronic conditions, such as laryngitis/pharyngitis, bronchitis, chronic cough, croup, etc.^5^^,^^6^^,^^7^ While substantial research has uncovered cellular diversity of the lower bronchopulmonary airways,^8^ comprehensive characterization of epithelial cell populations and their transcriptomic profiles within the lower pharyngeal and laryngeal regions, extending distally toward the tracheobronchial carina, remains limited. Given that respiratory diseases frequently exhibit regional specificity in their clinical manifestation, understanding epithelial cell heterogeneity at these specific anatomical locations is critical.

Research on the proximal conducting tracheal airway has identified significant cellular heterogeneity and revealed diverse roles in maintaining homeostasis^8^^,^^9^^,^^10^^,^^11^ and facilitating repair.^3^^,^^8^^,^^10^^,^^11^^,^^12^ Current evidence suggests that the larynx plays a significant role in shaping host immunity and thus mediates respiratory health and disease.^13^ Yet, the laryngeal transcriptome remains relatively underexplored at the single cell level.^13^ Moreover, previous studies have predominantly focused on single-organ analyses, with no investigations to date that comprehensively examine both the trachea and larynx in tandem. While commonly known cell types such as basal, ciliated, mucous/goblet, and club cells are widely present in mucosal regions of the trachea and portions of the larynx (e.g., superior vocal fold region and the inferior laryngeal aspect of the epiglottis), their transcriptomic profiles have not been fully characterized to determine whether significant differences exist between these anatomically distinct areas.

Submucosal glands (SMGs) are primarily localized within the larynx, with a smaller reservoir situated in the ventral proximal trachea. These glands play a pivotal role in airway innate immunity by regulating the secretion of antimicrobial peptides and mucins essential for mucosal defense.^14^^,^^15^^,^^16^ Additionally, recent studies have identified rare cell types with unique chemosensory and immune-mediated functions, including neuroendocrine cells,^17^ tuft cells,^18^^,^^19^ and region-specific cells such as hillock structures.^20^ For example, tuft cells, known as brush cells in the trachea and solitary chemosensory cells in the nasal, pharyngeal, and laryngeal epithelium, exhibit dual roles in immune and chemosensory effector functions.^19^^,^^21^^,^^22^^,^^23^^,^^24^ Neuroendocrine cells function as specialized sensory epithelial cells that protect the airways by releasing ATP to activate sensory neurons, triggering critical reflexes such as swallowing and expiration.^17^ Furthermore, unique tracheal hillock structures have been identified as injury-resistant reservoirs with distinct regenerative potential.^20^

With the variations in cell spatial patterning and the functional diversity of cell types, we hypothesized that the larynx and trachea possess unique, organ-specific transcriptomic profiles reflective of their specialized cell populations and tissue microenvironments. To analyze the cell diversity, we generated a comprehensive dataset covering the pharyngolaryngeal-to-tracheobronchial axis and established a single-cell atlas for the mouse adult upper respiratory epithelium. Our single-cell RNA-seq (scRNA-seq) analysis revealed 18 cell types across three spatially distinct compartments; (1) *Tmprss11a*+ stratified squamous epithelium (SSE) of the larynx, (2) *Nkx2-1*+ pseudostratified epithelium of the trachea, and (3) *Dmbt1+* SMG epithelium. Our dataset is rich with cell diversity, including abundant cell types, club (33%), basal (29%), and mucous-2-SMG (11%), as well as rare, specialized cell types, including mucous-larynx (1%), tuft (0.5%), and neuroendocrine (0.1%) cells. We further separated basal cell transcriptomic signatures and gene-ontology analysis based upon respective compartment (trachea-dorsal:*Trp63+Cav1+),* (trachea-ventral:*Trp63+Tgm2+),* (larynx:*Trp63+Igfbp2+Tmprss11a+*), and (SMG:*Trp63+Acta2+)*. This analysis of basal cells, paired with regional profiling, highlighted a unique expression profile of Keratins and established 21 total expressed genes along the proximal-distal and basal-luminal axes. Most notably, previously identified “stress-induced” Keratins, KRT6A and KRT17, were found to be present during homoeostasis, with expression in all basal cells as well as luminal squamous cells. Additionally, our luminal cell analysis identified three distinct types of club cells, with higher levels of *Scgb1a1*+ and *Scgb3a2+* expression in more distal compared to proximal airway regions. We also identified mucous-producing cells in the larynx, distinguished by regional gene expression and distinct cellular morphology, specifically *Il1a*+ squamous cells and *Kcnj16*+ cuboidal cells. Lastly, we profiled diversity of SMG cells, highlighting *Nkx3-1+* expression exclusive to mucous-1, mucous-2, and serous-acini cells, with ACTA2+ basal myoepithelial cells exhibiting a gene profile (*Ntng1, Ntn4, Slit3, Ntrk3, Cxcl12, Cxcl14*) linking potential neuroimmune strategies. Our single-cell transcriptomic analysis with *in situ* validations provides key insights into the transcriptomic and cellular heterogeneity of the upper airway and serves as a valuable atlas for hypothesis-driven work into responses to environmental insults, genetic mutations, and infectious diseases.

## Results

### Single cell transcriptomic analysis of the upper respiratory epithelium reveals compartmental and cellular heterogeneity

**Figure 1:**
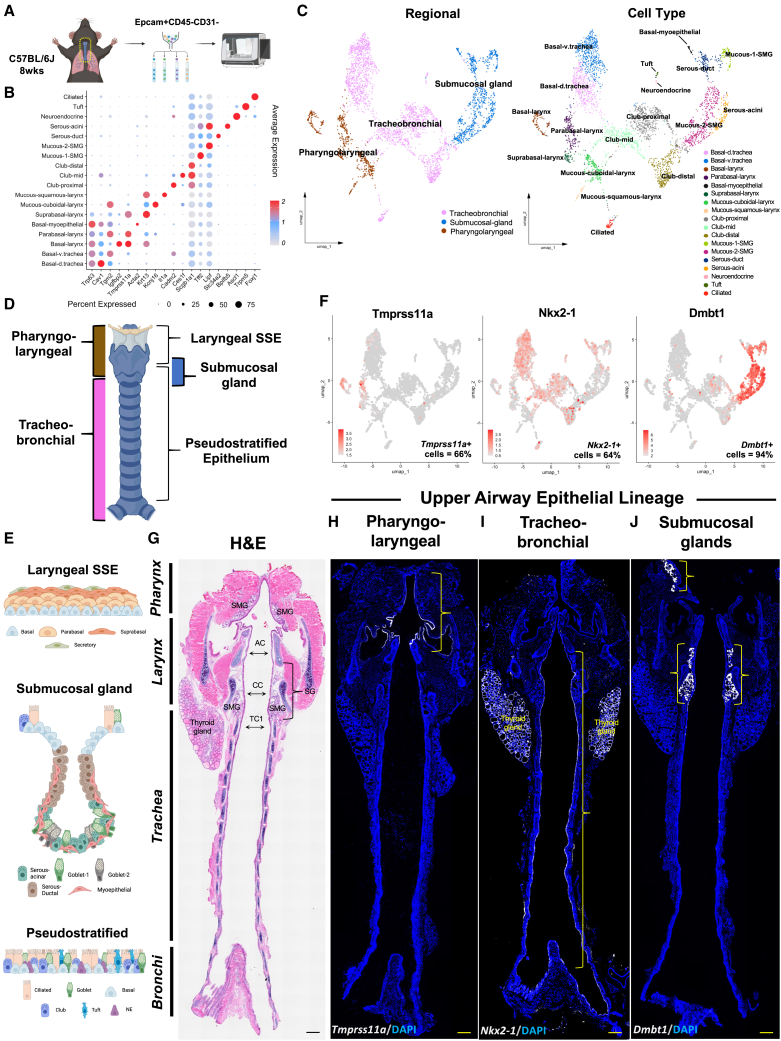
scRNA-seq of the upper respiratory epithelium reveal compartmental and cellular heterogeneity (A) Schematic illustration of cell isolation and scRNA-seq workflow. (B) Dotplot showing top markers for each cluster. Using default Seurat^25^ dotplot settings (Methods), percentage expressed was plotted from 0% to 75% detected and the color bar shows the average of scaled normalized expression values across cells in a given cluster. (C) UMAP plots of integrated upper airway scRNA-Seq data from adult WT B6 mice (*n* = 8 biological repeats, 4 male, 4 female). (D and E) Schematic illustration of macro-anatomical regions of the upper airway with compartmental epithelial cell type diversity. (F–J) Feature plots with RNAscope validation of *Tmprss11a* (pharyngolaryngeal) (H), *Nkx2-1* (tracheobronchial) (I), *Dmbt1* (seromucous glands) (J) exhibiting pan-epithelial regional markers in coronal sections. (G) H&E-stained upper airway. DAPI is in blue. All images 20× magnification. Scale bar represents 500 μm. H&E, hematoxylin and eosin; AC, arytenoid cartilage; CC, cricoid cartilage; TC, tracheal cartilage; SSE, stratified squamous epithelium; SG, subglottis; SMG, submucosal gland.

To explore epithelial cellular diversity of the upper respiratory epithelium, we started by using scRNA-seq to profile the pharyngolaryngeal-to-tracheobronchial axis. We isolated and pooled epithelial cells from pharynx to bronchi regions of adult wildtype C57BL/6 mice (*n* = 4 males, 4 females), and applied them to 10x Chromium for scRNA-seq (Figure 1A). Following quality control (Figure S1A), 3,617 epithelial single cells were recovered. Applying unsupervised clustering with Seurat default package,^25^ followed by supervised annotation using known markers (Figures S1A–S1D),^9^^,^^12^^,^^13^^,^^16^^,^^26^ we identified 18 cell types delineated with top markers (Figure 1B; Tables S1–S3). These 18 cell types segregate into three compartments; pharyngolaryngeal*,* tracheobronchial, and SMG (Figure 1C). In further analysis of the pharyngolaryngeal stratified squamous epithelium (SSE), we identified *Tmprss11a* as a marker for this compartment (Figure 1H). The tracheobronchial pseudostratified epithelium was marked by *Nkx2-1,* a known marker that was region-specific, aside for its expression in the thyroid epithelium (Figure 1I). In the SMG epithelia, we identified *Dmbt1* as a region-specific marker, which was expressed at a high level with negligible expression in a few club cells in the proximal trachea and subglottic surface epithelium (Figure 1J). We validated these regional expression profiles using RNAscope on coronal sections along the pharyngolaryngeal-to-tracheobronchial axis, defining the spatial localization of the pharyngolaryngeal (*Tmprss11+),* tracheobronchial (*Nkx2-1)*, and SMG (*Dmbt1+)* compartments (Figures 1D–1J; Figures S1E–S1G). Additionally, our analysis quantified the percentage of cells expressing each marker within their respective compartments, confirming enrichment of *Tmprss11a* expression in 66% of pharyngolaryngeal cells, *Nkx2-1* in 64% of tracheobronchial cells, and *Dmbt1* in 94% of SMG cells (Figure 1F; Figure S1G). These expression patterns elucidate the transcriptomic specificity for regional targeting of these epithelial compartments.

### Genome-wide profiling identifies signatures of the stratified squamous epithelium (SSE) along the basal-luminal axis

**Figure 2:**
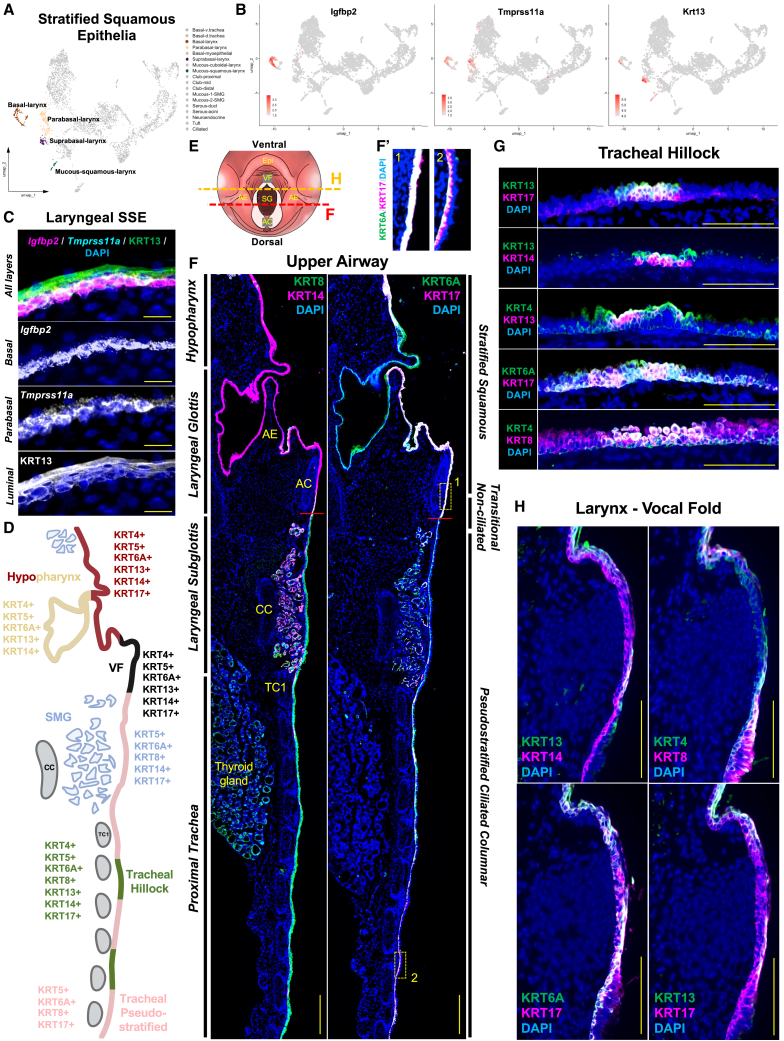
Genome-wide profiling revealed signatures of stratified squamous epithelium along basal-luminal axis with extensive Keratin diversity along proximal-distal axis (A) Integrated UMAP plot highlighting laryngeal surface epithelial cells. (B and C) Feature plots with RNAscope validation of basal-to-luminal canonical markers for laryngeal SSE. (D) Schematic illustration of KRT diversity along the proximal-to-distal upper respiratory axis. (E) Schematic illustration of cranial view of larynx to display coronal sections (red/yellow dashed lines) attained for analysis. (F and F′) Immunofluorescent coronal serial sections with insets of dorsal upper airway epithelium displaying regional KRT expression along various epithelium subtypes. Red line indicates transition zone from SSE to pseudostratified. (G and H) Diversity of KRT expression to SSE of tracheal hillocks and laryngeal VF tissue. DAPI is in blue. All images 20× magnification. Scale bar represents 100 μm (C, G, and H) and 500 μm (F). Epi, epiglottis; AE, aryepiglottic fold; SG, subglottis; AC, arytenoid cartilage; CC, cricoid cartilage; TC, tracheal cartilage; SSE, stratified squamous epithelium; KRT, keratin; SMG, submucosal gland.

The pharynx and larynx are mostly lined with SSE, which caudally extends to the infraglottic vocal fold (VF),^27^^,^^28^ and is also present in hillock regions of the trachea.^20^ This contrasts from the subglottis, trachea, and bronchi, which are lined with pseudostratified epithelium (Figures 1D, 1E, and 1G).^27^^,^^28^ The multi-layered SSE serves to protect the underlying tissues from mechanical and chemical damage, pathogens, and dehydration.^20^^,^^29^ Given the unique architecture, we hypothesized that this specialized SSE tissue would exhibit a basal-to-luminal coding pattern. Using SSE of the larynx as an example, we identified four cell clusters representing cell types along the basal-luminal axis: basal, parabasal, suprabasal/luminal, and mucous (Figures 2A and 2B). To explore the regional expression profile *in situ*, we utilized RNAscope and discovered that *Igfbp2* is exclusively expressed in the basal layer, *Tmprss11a* expressed in the basal and parabasal layers, and KRT13 protein distribution extending from basal to suprabasal with increased expression to differentiated luminal cells (Figures 2B and 2C; Figure S1I). We also identified populations of mucous-producing cells dispersed throughout the pharyngolaryngeal apical epithelium, exhibiting top marker *Il1a* expression (Figure 1B; Figure S2C). Together, these data define the compartmental specificity of squamous epithelia and unique expression profiles during terminal differentiation.

### Systematic profiling of Keratin code along the proximal-distal and basal-luminal axes

Keratins are essential for maintaining the structure and function of epithelial cells, playing pivotal roles in homeostasis, tissue repair, and disease pathogenesis.^30^^,^^31^^,^^32^^,^^33^^,^^34^^,^^35^^,^^36^^,^^37^^,^^38^ Specific Keratins are often markers for specialized epithelial cells, e.g., KRT13+KRT14+ for VF SSE and KRT4+KRT13+ for tracheal hillocks.^8^^,^^20^^,^^39^ However, the cell type-specific expression and spatial pattern of diverse Keratins have not been systematically characterized in the upper respiratory epithelium. We started with exploring Keratin gene expression in our scRNA-seq dataset. Among the 63 known Keratin genes, expression of 21 of them were detected in the upper respiratory epithelium (Figure S3). Along the proximal-distal axis, we categorized the “Keratin code” based on scRNA-seq data (Figure 2D). We validated the data using immunofluorescence staining of key Keratins on serial sections (Figures 2E–2H; Figures S1H and S1I). The proximal-distal axis along the surface epithelium was defined by complementary KRT8 and KRT14 expression (Figure 2F; Figure S1H). Specifically, KRT8 was primarily detected in tracheal pseudostratified epithelium, SMG epithelium, and thyroid gland epithelium, while KRT14 was detected primarily in laryngeal SSE, myoepithelial cells of SMG, and a subset of basal cells of the proximal trachea (Figures 2F–2H; Figure S1H). KRT6A and KRT17, known as “stress-induced” Keratins,^30^^,^^31^^,^^32^^,^^34^ were both highly expressed in all three compartments, defined by exclusive basal cell expression in pseudostratified and SMG epithelium, albeit, enriched in both basal and luminal cells in laryngeal SSE and tracheal hillocks (Figures 2F–2H; Figure S1H). Interestingly, and unique to the mouse, the lingual side of the aryepiglottic fold, which transitions ventrally into the epiglottis, contains cornified epithelium which did not express KRT17 (Figures 2D and 2F; Figure S1H). KRT4 and KRT5 were expressed across all layers of the SSE; however, KRT5 expression was particularly enriched within basal cells (Figure S1I). We also compared the Keratin code in the VF and tracheal hillock squamous epithelium, two upper respiratory regions that are under constant mechanical insults. Keratin expression was abundant in both of these tissues with only subtle differences. Tracheal hillocks exhibited a Keratin coding pattern defined by basal markers (KRT4+KRT5+KRT6A+KRT14+KRT17+) and luminal markers (KRT4+KRT6A+KRT8+KRT13+) (Figures 2F and 2G). VF SSE displayed identical basal marker Keratin expression to hillocks, and with identical luminal markers excluding KRT8 to the VF medial edge (Figures 2F, 2G, and 2H). Together, these data reveal a complex Keratin code in the upper respiratory epithelium (Figure 2D; Figure S3).

### Basal cells exhibit transcriptomic regional diversity with unique functional signatures

**Figure 3:**
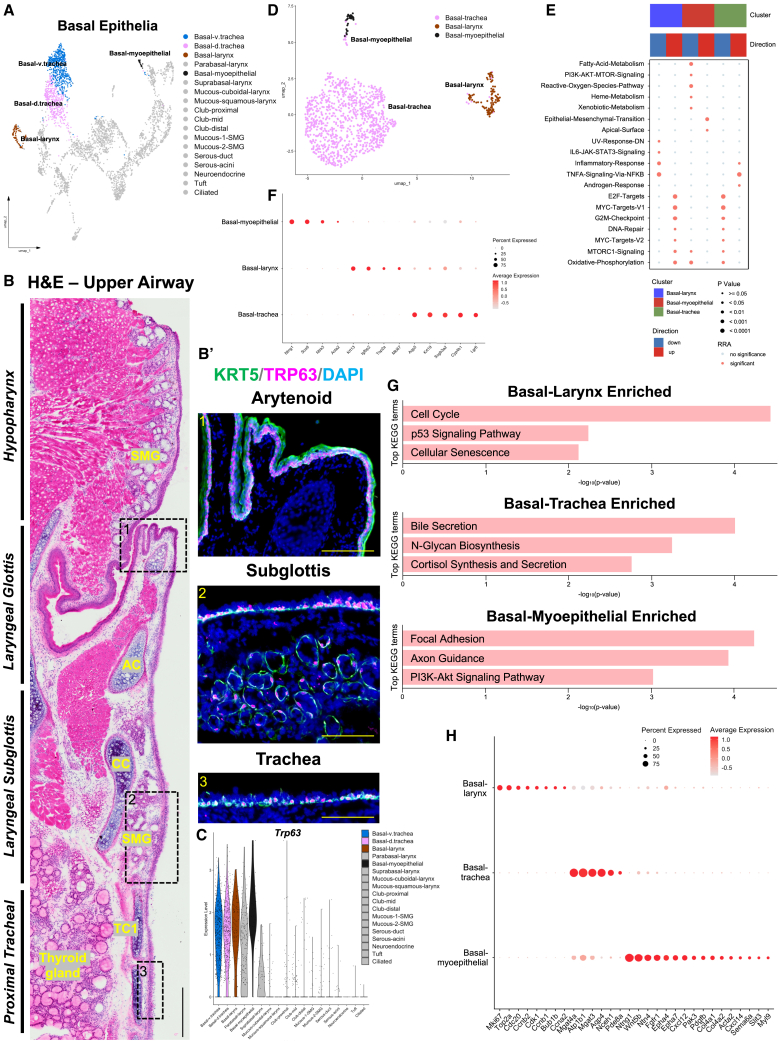
Basal cells exhibit transcriptomic regional diversity with unique functional signatures (A) Integrated UMAP plot highlighting basal epithelial cells. (B and B′) H&E-stained upper airway with immunofluorescence exhibiting regional KRT5+TRP63+marked basal cells. (C) Violin plot exhibiting cell types that express canonical basal cell marker *Trp63*. (D) Integrated UMAP of basal cell subset. (E) irGSEA hallmark pathways analysis of DE genes across our three basal populations. (F) DotPlot showing top DE genes comparing basal-larynx (*Krt13, Igfbp2, Top2a* and *Mki67*) to basal-trachea (*Aqp5, Krt19, Scgb3a2, Cyp4b1* and *Lgr6*) to basal-myoepithelial (*Ntng1, Sox9, Ntrk3* and *Acta2*) in our basal cell subsetted scRNA-seq dataset. (G) Gene ontogeny (GO) enrichment analysis displaying top KEGG pathway terms enriched in each basal cell. (H) DotPlot showing top DE genes associated with functional enrichment analysis. DAPI is in blue. All images 20× magnification. Scale bar represents 100 μm. H&E, hematoxylin and eosin; KRT, keratin; AC, arytenoid cartilage; CC, cricoid cartilage; TC, tracheal cartilage; SMG, submucosal gland.

Basal cells pave the entire upper respiratory epithelium, from pharynx to bronchi marked by high expression of the putative progenitor cell marker *Trp63* (Figure 3A–3C). To investigate the distinct patterning of these cells, we performed immunofluorescence and assessed *Trp63* expression in three compartments. Our analysis revealed multi-layered TRP63+ basal cells in the SSE of the larynx, single-layered basal cells in the trachea and subglottis and distributed basal cells in the SMG (Figures 3B and 3B′). To further explore functional differences based on these anatomic locations, we isolated out basal populations *in silico* (Figure 3D) and performed differential expression (DE) analysis (p_adj = <0.01, avg_logFC = 2.5), comparing across basal subtypes (Figure 3E), followed by Gene Ontogeny (GO) and irGSEA hallmark pathways analysis (Figures 3E–3H; Figure S2A). The irGSEA hallmark pathway analysis, based on DE genes across all basal groups, found unique profiles within the basal-larynx, basal-myoepithelial, and basal-tracheal cell populations. Basal-larynx cells were enriched for G2M-Checkpoint, MYC-Targets-v1/v2, and DNA-Repair (*Krt13, Igfbp2*, *Top2a*, and *Mki67*). Basal-myoepithelial cells were enriched for Epithelial-Mesenchymal-Transition and Apical-Surface (*Ntng1, Sox9, Acta2*, and *Ntrk3*). Lastly, basal-tracheal cells showed enrichment for Inflammatory-Response and TNFα-Signaling-via-NF-κB (*Aqp5, Krt19, Scgb3a2, Cyp4b1, Lgr6*) (Figures 3E and 3F). To rigorously assess these transcriptomic signatures, we conducted Gene Ontology (GO) enrichment and identified relative DE genes associated with KEGG pathway terms (Figures 3G and 3H; Figure S2A; Table S4). Basal-larynx cells showed top enriched pathways related to Cell Cycle, p53 Signaling Pathway and Cellular Senescence (*Cdc20*, *Ccna2*, *Ccnb2*, *Ccnb1*, *Cdk1*, and *Bub1b*). Basal-tracheal cells exhibited enrichment for Bile Secretion, N-glycan Biosynthesis, and Cortisol Synthesis and Secretion (*Nceh1*, *Aqp4*, *Atp1b1*, *Mgat4a*, *Mgat3*, and *Pde8a*). Meanwhile, basal-myoepithelial cells showed enrichment for Focal Adhesion, Axon Guidance, and PI3K-Akt Signaling Pathway (*Ntng1*, *Epha4*, *Epha7*, *Sema6a*, *Cxcl12*, *Wnt5b*, *Ntn4*, *Slit3*, *Pak3*, and *Myl9*) (Figures 3G and 3H). For a comprehensive list of GO enrichment and irGSEA hallmark pathways analysis of DE genes across all epithelial cell types refer to Table S3 and Figure S4, respectively. This analysis highlights the distinct transcriptional and functional specialization of basal cell populations across the upper respiratory epithelium, providing a foundation for understanding their unique roles in homeostasis, repair, and regional adaptation.

Basal cells are progenitor cells that are critical for repair and regeneration of the epithelium following injury by differentiating into specialized cell types needed for tissue integrity. To compare the potential differentiation pathways in each of the basal cell types, we performed single cell trajectory analysis (Monocle 3). *In silico,* we selected cell clusters to only include each basal cell population (i.e., basal-larynx, basal-trachea, and basal-myoepithelial) alongside the differentiated cell types in the corresponding region (Figures S5A–S5C). The basal-larynx trajectory revealed a direct lineage progression from basal to parabasal to suprabasal cells, subsequently leading to differentiated cell populations including mucous, ciliated, and club cell identities. The basal-trachea trajectory displayed a sequential progression through club-mid, club-proximal, and club-distal stages, followed by mucous-related lineages, with ciliated cells representing the most distal point in pseudotime. In comparison, basal-myoepithelial cells followed an SMG lineage trajectory progressing through mucous-2, serous-acini, serous-duct, mucous-1, ending in ciliated cells (Figures S5A–S5C). Our data highlights that basal cells, despite their morphological similarity, exhibit transcriptional heterogeneity primed for shared but also distinct roles across different epithelial compartments.

### Submucosal gland cell heterogeneity as revealed at single cell resolution

**Figure 4:**
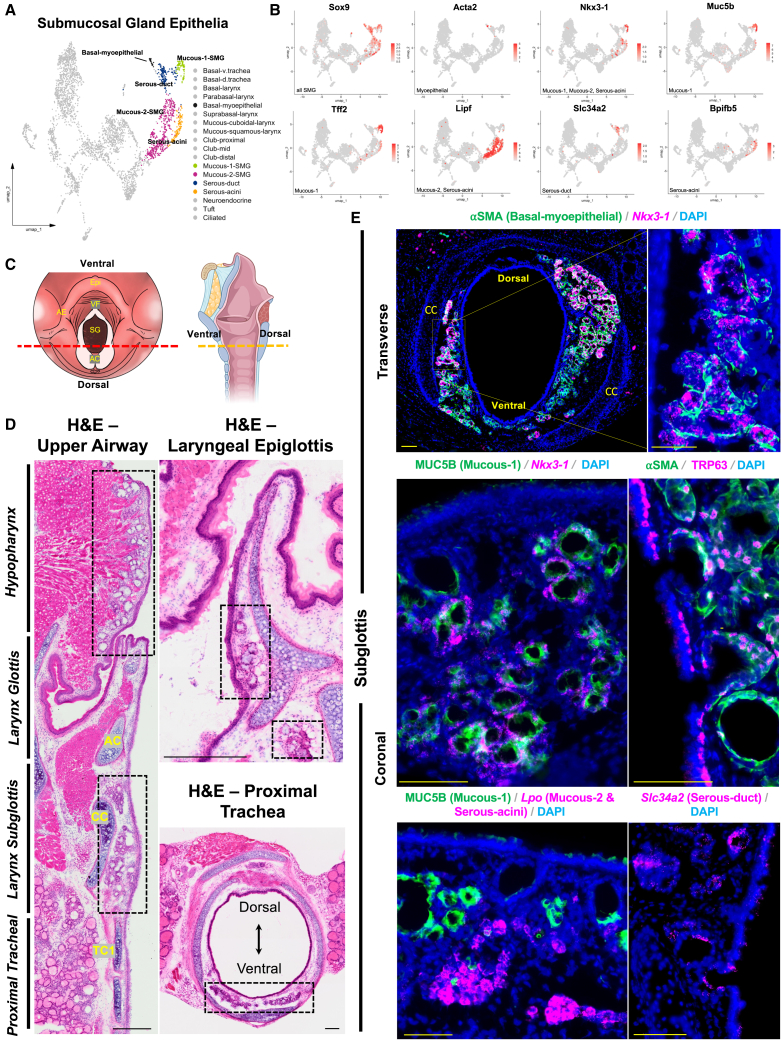
Submucosal gland cell heterogeneity as revealed at single cell resolution (A) Integrated UMAP plot highlighting SMG epithelial cells. (B) Feature plots of canonical markers for SMG epithelial populations. (C) Schematic illustration of cranial and sagittal view of larynx to display coronal and transverse sections (red/yellow dashed lines) attained for analysis. (D) H&E-stained upper airway displaying SMG compartments (dashed black boxes). (E) Immunofluorescence transverse and coronal sections of SMGs displaying cell type canonical markers and unique transcription factor *Nkx3-1*. DAPI is in blue. All images 20× magnification. Scale bar represents 100 μm. H&E hematoxylin and eosin. Epi, epiglottis; AE, aryepiglottic fold; SG, subglottis; AC, arytenoid cartilage; CC, cricoid cartilage; TC, tracheal cartilage.

We sought to explore the heterogeneity of the murine SMG compartment, which has largely been unexplored at the single-cell level.^3^ Our scRNA-seq revealed the expected rich diversity of differentiated cell types in the SMG, and also uncovered markers for each population (Figures 4A and 4B). In the mouse upper respiratory tract, SMG were found in the pharynx, rostral laryngeal side of the epiglottis, subglottis, and proximal ventral trachea (Figures 4C and 4D). Based on our scRNA-seq data, these SMG epithelial cells segregate into multiple populations, including mucous-1 (*Muc5b, Tff2*), mucous-2 (*Lipf*), serous-duct (*Slc34a2*), serous-acini (*Bpifb5, Lipf*), and basal-myoepithelial cells (*Acta2, Trp63*), each with a distinct transcriptional profile (Figures 4A and 4B). *Sox9* transcripts were also abundant in all SMG cells, with additional expression in the tuft cell population. Interestingly, RNAscope validation showed that the transcription factor *Nkx3-1* was expressed in mucous-1, mucous-2, and serous-acini cells (Figure 4E), suggesting a unique role in regulating mucous producing cell fate. ⍺SMA+TRP63*+* basal-myoepithelial cells were found primarily in the distal acinar compartment and did not overlap with *Nkx3-1* expression (Figure 4E). Notably, all SMG compartments, including the epiglottic, subglottic, hypopharyngeal, and tracheal regions, exhibited similar expression patterns for known cell types, suggesting a shared transcriptional signature and conserved functional role across these anatomically distinct glandular populations (Figure S6). However, further lineage-tracing studies would be needed to formally determine whether distinct progenitor pools contribute to regional SMG formation. Together, these data delineate both shared and distinct signatures of murine SMG cell types.

### Luminal cell heterogeneity as revealed at single cell resolution

**Figure 5:**
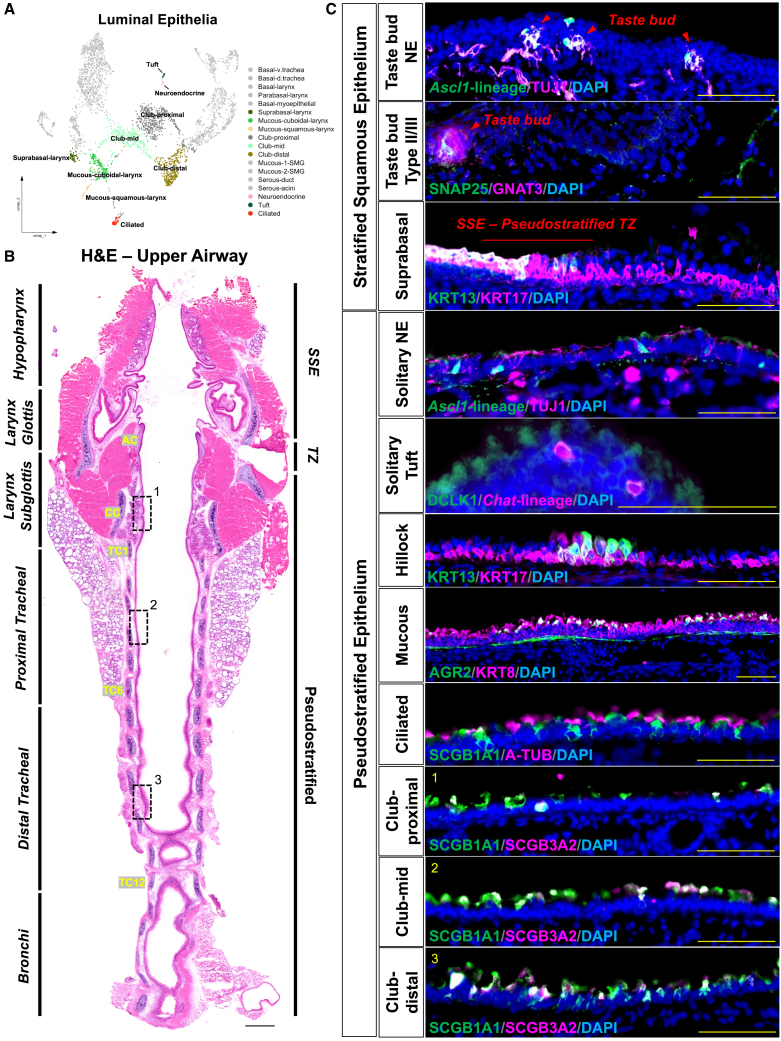
Luminal cell heterogeneity along pharyngolaryngeal-to-tracheobronchial axis revealed at single cell resolution (A) Integrated UMAP plot highlighting luminal epithelial cells. (B) H&E-stained upper airway displaying anatomic regions of interest with dashed black boxes representing localized regions for club-proximal, club-mid, and club-distal immunofluorescent corresponding images. (C) Immunofluorescence images exhibiting unique markers for luminal cell types of the upper airway. DAPI is in blue. Images are either 20× or 40× magnification. Scale bar represents 100 μm (C) and 500 μm (B). H&E, hematoxylin and eosin; AC, arytenoid cartilage; CC, cricoid cartilage; TC, tracheal cartilage; TZ, transition zone; NE, neuroendocrine; SSE, stratified squamous epithelium.

We further analyzed our scRNA-seq dataset to reveal the diversity of luminal cell populations along the pharyngolaryngeal-to-tracheobronchial axis (Figures 5A–5C). Luminal cell types located to laryngeal SSE include suprabasal cells (KRT13, KRT17), mucous-producing cells (*Il1a, Kcnj16*), and taste bud complexes which include innervated neuroendocrine (*Ascl1*), type II (GNAT3) and type III (SNAP25) taste cells (Figure 5C; Figures S2B and S2C). Solitary neuroendocrine cells were also found within the highly innervated subglottic epithelium and decreased in number along the proximal-distal axis toward the tracheal epithelium (Figure 5C; Figure S2B). Luminal cell types located in the trachea epithelium include club (SCGB1A1, SCGB3A2), mucous (AGR2), ciliated (A-TUB), solitary tuft (DCLK1, *Chat*)*,* and clustered hillocks (KRT13, KRT17). Ciliated cells were found throughout the pseudostratified epithelium of the trachea and in focal regions within the laryngeal epithelium, such as the superior VF and rostral lingual side of the epiglottis (Figure S2C). Additionally, mucous-1 cells, identified by the classic goblet cell marker AGR2,^40^ were detected in the tracheal surface epithelium by immunofluorescence (Figure 5C) and were notably enriched within the *Dmbt1+* SMG compartment (Figure S2D). We found heterogeneity within club cells along the tracheobronchial axis which we termed club-proximal, club-mid and club-distal, with subtypes showing unique regional transcriptomic signatures and cell abundance (Figure 5A; Figures S2E–S2K).

To directly compare DE genes, we took a similar approach as our basal analysis and isolated club cells *in silico*, and subclustered the population (Figure S2I; Table S5). Notably, distal club cells expressed the highest levels of *Scgb1a1* and *Scgb3a2* (Figure 1B; Figures S2E–S2K), which was validated by pairwise DE comparison analysis (p_adj = <0.05, LogFC = 1) between club-proximal and club-distal (Figure S2H; Table S5). *Slc6a15* emerged as the top marker for club-proximal population across all cell types (Figure 1B), which also showed elevated expression of *Cadm2* (Figure S2J; Table S1), a key defining marker expressed in laryngeal club cells.^13^ Immunofluorescence confirmed an increase in SCGB1A1+SCGB3A2+ club cell abundance to the distal trachea and extrapulmonary bronchi compared to proximal regions (Figure 5C; Figures S2F and S2G). GO enrichment and pathway analysis revealed the top KEGG terms for club-proximal cells, including Ras Signaling Pathway (*Angpt1*, *Abl1*, *Gab2*, and *Gnb5*), and Calcium Signaling Pathway (*Chrm3*, *Gna14*, *Erbb4*, and *Tacr1*). In contrast, club-mid cells showed enrichment for KEGG terms related to Inflammatory Mediator Regulation of TRP Channels (*Camk2d, F2rl1, Calml3*) and Mucin-Type O-Glycan Biosynthesis (*Galntl6* and *B4galt5*). Finally, club-distal cells were enriched for KEGG terms linked to Ribosome function and Coronavirus Disease (*Mrpl4*, *Rps26*, *Mrpl2*, *Rps19*, *Rps7*, *Rpl36al*, *Rplp0*, and *Rps12*) (Figure S2K; Table S5). Together, our analysis suggests potential regional-specific roles for club cells, where club-proximal cells located in the highly innervated laryngeal subglottis may play a prominent role in xenobiotic detection, while club-mid and club-distal cells localized to the trachea are primarily involved in secretory/mucin pathways and metabolic processes.

## Discussion

To date, research has established diverse heterogeneity across the cellular landscape of the unified respiratory epithelium,^8^^,^^13^ offering an initial basis for its complexity. Building on these investigations focused on individual organs, here, we present a comprehensive analysis of the single cell landscape in the murine upper airway, encompassing the pharyngolaryngeal-to-tracheobronchial axis. We defined three spatially distinct tissue compartments *Tmprss11a*+ pharyngolaryngeal, *Nkx2-1*+ tracheobronchial, and *Dmbt1*+ SMG epithelium, comprised of 18 transcriptionally unique cell types. This comprehensive regional atlas provides a critical framework for future studies aimed at understanding cellular dynamics in airway health, disease progression, and repair mechanisms.

Our analysis revealed a robust population of TRP63+ basal cells, characterized by regional transcriptomic diversity and distinct functional signatures. Consistent with prior studies demonstrating basal cell subpopulations in dorsal versus ventral tracheal regions,^12^ we identified a dorsal-enriched population (*Trp63+Cav1+*) and a ventral-enriched population (*Trp63+Tgm2+*) of tracheal progenitors. In the larynx, basal cells exhibited unique enrichment for *Igfbp2*, with a concomitant reduction in *Tmprss11a* expression among SSE progenies. While *Tmprss11a* has been implicated in epithelial integrity and protease activity,^41^^,^^42^ its specific role in the pharyngolaryngeal compartment remains unclear, highlighting the need for future targeted work. Laryngeal SSE display higher cellular turnover compared to the predominantly quiescent pseudostratified epithelium.^20^^,^^43^ Comparative analyses between basal cells from the larynx and trachea revealed elevated expression of proliferative markers in laryngeal basal cells suggestive of key roles in tissue repair, while tracheal basal cells enriched for markers associated with secretory function indicate their contribution to mucosal defense. These findings highlight distinct regional specialization of basal cell populations between the larynx and trachea, underscoring their functional divergence in maintaining epithelial integrity.

We also explored Keratin gene expression within our scRNA-seq dataset with the goal of establishing a comprehensive Keratin profile of the upper respiratory epithelium. Systematic profiling identified a total of 21 represented Keratin genes, delineating a distinct expression code along both the proximal-distal and basal-luminal axes. Among these, we focused on the “stress-induced” Keratins, KRT6A and KRT17. While previous studies have reported ectopic expression of KRT6A and KRT17 in epithelium following wound healing^34^ or in the context of viral persistence,^31^^,^^32^ our findings reveal widespread expression under homeostatic conditions. Specifically, KRT6A+KRT17+ epithelium was detected in basal cells of the trachea and SMG, as well as in both basal and luminal cells of the laryngopharynx and tracheal hillocks. We also observed restricted expression of KRT8 to pseudostratified surface epithelium and KRT14 to squamous surface epithelium of the pharyngolarynx, delineating a distinct proximal-distal axis of epithelial differentiation. KRT13 at the protein and gene level marked suprabasal (i.e., luminal) SSE, however, was also modestly expressed in our basal-larynx population. Recent work has reported KRT13 expression in basal cells of hillocks.^20^ Immunofluorescent validation found hillocks expressed a near identical Keratin code as VF SSE; thus, we suspect that hillocks share a sufficiently similar transcriptome with differentiated SSE, leading them to be grouped into our suprabasal-larynx population. To date, no studies have investigated airway SSE of both the larynx and trachea within one dataset at single cell resolution. These findings highlight previously uncharacterized Keratin dynamics across epithelial subtypes, providing new insights into airway squamous epithelial differentiation and potential shared transcriptional profiles with specialized structures like hillocks.

Next, we sought to characterize the cellular diversity of an underexplored, yet essential tissue compartment, the SMG. SMG cells play critical roles in upper airway immune defense, actively secreting mucus and antimicrobial byproducts to defend against pathogens and irritants, and with unique basal-myoepithelial cells.^15^^,^^44^ We identified *Nkx3-1* transcription factor, uniquely expressed in mucous-1, mucous-2, and serous-acini cells. *Nkx3-1*, widely recognized as a prostate epithelium-specific marker, plays a key role in the development of the testes and prostate and has been implicated in prostate cancer.^45^^,^^46^ Given its cell-specific expression and well-established roles in development, we hypothesize that this transcription factor may influence the cell fate of mucin-producing cells in the SMG, potentially serving as an effective candidate for disease modeling. The subglottic epithelium shares a close anatomical and functional relationship with adjacent SMG. Notably, *Acta2+* myoepithelial cells within SMG function as reserve stem cells capable of regenerating the airway surface epithelium.^3^^,^^47^^,^^48^ In salivary glands, myoepithelial cells have been identified as key sources of neurotrophin signaling following irradiation-induced damage.^49^ When comparing basal-myoepithelial cells from the larynx and trachea, we observed increased expression of genes associated with axon guidance (*Ntng1*, *Wnt5b*, and *Ntn4*), cytokine signaling (*Cxcl12* and *Cxcl14*), and neurotrophic receptors (*Ntrk2* and *Ntrk3*). The *Cxcl12-Cxcr4* axis is a well-characterized signaling pathway with pleiotropic roles in immune responses, viral infections, and cancer.^50^^,^^51^ Recent studies have further demonstrated that *Cxcr4* is upregulated following influenza infection, modulating neutrophil phagocytic activity, ligand-specific migration, and the induction of neutrophil extracellular traps.^52^ Based on our findings, we propose that basal-myoepithelial cells may play a critical role in injury repair and immune response mediated by the *Cxcl12/14-Cxcr4* axis. In addition, we detected upregulation of CHAT muscarinic receptors (*Chrm1* and *Chrm3*) in basal-myoepithelial cells, suggesting potential crosstalk with cholinergic-expressing neurons in the airways. These findings highlight the intimate relationship between basal-myoepithelial cells and innervating neurons and suggest a critical role for these cells in mediating neuroimmune interactions that contribute to airway homeostasis and disease.

The upper respiratory epithelium of the larynx and trachea contains several shared luminal cell types, including club, mucous/goblet, and ciliated cells. However, the transcriptomic differences between these regions remain incompletely characterized. To address this, we hypothesized that distinct transcriptomic profiles would emerge based on anatomical localization. Our analysis identified three transcriptionally distinct populations of club cells, with higher levels of *Scgb1a1*+ and *Scgb3a2+* expression in more distal compared to proximal regions along the upper airway axis. Club cells, defined by secretoglobin family genes (*Scgb1a1*, *Scgb3a1*, and *Scgb3a2*), have been shown to exhibit functional heterogeneity in previous studies.^8^^,^^53^^,^^54^ A unique club-proximal population, exclusively expressing the defining laryngeal club cell marker *Cadm2*, was localized to the larynx.^13^ Additional populations, designated as club-mid and club-distal, were restricted to the proximal trachea and distal trachea/extrapulmonary bronchi, respectively. Notably, laryngeal club cells, which have only recently been described, exhibit both squamous and cuboidal morphologies,^27^ and are implicated in detoxification, repair, regeneration, and anti-inflammatory processes.^13^^,^^54^^,^^55^ We identified rare mucous-producing cell populations within the larynx, characterized by squamous or cuboidal morphology and elevated expression of mucin-related genes (*Muc1*, *Muc4*, *Muc13*, and *Muc20*). These cells were further distinguished by distinct top markers: *Il1a* for squamous-type and *Kcnj16* for cuboidal-type populations. Notably, *Il1a*+ cells exhibited strong expression of *Duoxa2*, an enzyme essential for thyroid hormone synthesis and mucosal immunity.^56^ Additional genes highly expressed in this rare cell subset included interleukin signaling components (*Il1a*, *Il1r2*, and *Il1rn*), the transcription factor *Irf7*, and genes related to inflammatory responses and pathogen defense (*Isg15*, *Tnf*, and *Tnfrsf11b*). Collectively, the unique transcriptional signature of these cells suggests a specialized role in coordinating mucosal defense and immune responses to pathogens within the laryngeal epithelium.

In addition to commonly shared cell types, several rare yet functionally significant populations exist in the upper respiratory tract, including neuroendocrine cells, tuft cells, and ionocytes, each contributing uniquely to airway homeostasis and function. Our dataset identified both neuroendocrine and tuft cells; however, ionocytes were not detected. The relatively modest cell yield (3,617 cells) likely limited our capacity to detect rare or transient cell populations, such as ionocytes, transitional epithelial cells, or cells sensitive to processing-related loss.^8^ Solitary neuroendocrine cells expressed canonical markers (*Ascl1, Calca*)^8^^,^^57^ and exhibited elevated expression of *Cxcl13* and *Ngf*, with the highest abundance within the laryngeal subglottic epithelium. Neuroendocrine cells in the lung, known as specialized airway sensors, are critical for interacting with peripheral nerves to regulate immune responses to allergens.^58^^,^^59^ Tuft cells in our dataset were marked by classical tuft cell genes (*Pou2f3*, *Spib*, *Trpm5*, and *Dclk1*) along with genes associated with immune regulation (*Rgs13*, *Rgs14*, *Rgs21*, *Tnfrsf13b*, and *Il17rb*),*^13^* type II taste cell marker *(GNAT3)* and basal cell identity (*Lgr5*). Although limited cell numbers precluded identification of subpopulations, other studies have reported heterogeneity within tuft cells, including tuft-1 (*Pou2f3*) and tuft-2 (*Pou2f3*, *Gfi1b*, *Spib*, and *Sox9*) subtypes in the tracheal epithelium.^8^^,^^21^ Tuft cells have also been characterized as sources of IL-25, prostaglandins, leukotrienes, and acetylcholine,^19^^,^^22^^,^^23^^,^^24^^,^^60^^,^^61^ which are crucial for promoting innate type 2 immunity and protective neural reflexes.^62^ Our use of Chat^cre^;tdTomato lineage-marked tissue confirmed CHAT expression in tracheal tuft cells, supporting their acetylcholine-producing capacity and aligning with previous work.^18^^,^^63^ While our *in silico* analysis indicated that both mucous-1 and mucous-2 cells localize predominantly to the *Dmbt1*-positive SMG compartment, our immunofluorescence data suggest that a subset of mucous-1 cells also resides in the tracheal surface epithelium, aligning with previous work.^8^ These findings underscore the extensive heterogeneity of epithelial cells across multiple upper airway compartments, emphasizing their roles in defending against environmental insults and maintaining homeostasis.

The extensive transcriptomic and cell diversity of the upper respiratory tract enables the epithelium to perform a range of functions, from forming physical barriers and initiating immune responses to repairing tissue damage and regulating inflammation. Our findings establish a region-specific specialization of epithelial diversity and a detailed cross-organ single-cell atlas along the pharyngolaryngeal-to-tracheobronchial axis, offering valuable insights into the diverse cell populations that maintain airway health and resilience.

### Limitations of the study

While our single-cell transcriptomic atlas of larynx and trachea epithelium provides unprecedented insights into airway cellular heterogeneity, several constraints warrant acknowledgment. The limited biological replication (8 mice yielding 3,617 cells) potentially restricts our ability to capture rare cell populations, such as ionocytes, which typically constitute <1% of airway epithelium. Furthermore, our technical design impedes robust pseudo-bulk statistical comparisons, potentially affecting the confidence intervals of our differential expression analyses. Our focus on healthy adult murine tissue, while establishing a critical baseline, necessarily constrains immediate translation to developmental trajectories or pathological states. These limitations present clear opportunities for future research. Expanding the dataset with greater biological replication would enhance statistical power for detecting subtle transcriptional signatures. Additionally, extending this atlas to include developmental timepoints and disease models would significantly amplify its translational impact, particularly for respiratory conditions affecting the upper airways. Ultimately, this work serves as a foundational resource that invites expansion across physiological states to fully capitalize on its potential for understanding airway biology and pathology.

## Resource availability

### Lead contact

Further information and requests for resources and reagents should be directed to and will be fulfilled by the lead contact, Dr. Xin Sun (xinsun@health.ucsd.edu).

### Materials availability

This study did not generate new unique reagents.

### Data and code availability

Single cell RNA-seq data have been deposited at the Gene Expression Omnibus (GSE287495) and are publicly available as of the date of publication. All data generated supporting the findings of this study are available in the manuscript. Further information is available from the lead author upon reasonable request. This paper does not report original code.

## Acknowledgments

The authors thank Sun lab members for discussions. UCSD Microscopy Core was supported by NINDS-P30NS047101. This work was supported by grants NIH NHLBI R01 AT011676-01 (to X.S.), 1R01 HL160019-01 (to X.S.), and NIH NIDCD F32 DC021634-01 (to A.G.F.).

## Author contributions

Conceptualization: A.G.F. and X.S.; methodology: A.G.F.; investigation: A.G.F.; visualization: A.G.F.; funding acquisition: A.G.F. and X.S.; supervision: X.S.; writing – original draft: A.G.F.; writing – review and editing: A.G.F. and X.S.

## Declaration of interests

The authors declare no competing interests.

## STAR★Methods

### Key resources table

**Table:** 

REAGENT or RESOURCE	SOURCE	IDENTIFIER
**Antibodies**		

Rabbit, anti-KRT13	Abcam	Cat#ab92551; RRID: AB_2134681
Mouse, anti-KRT14	Millipore	Cat#CBL197; RRID: AB_2132747
Rabbit, anti-KRT17	Santa Cruz Biotechnology	Cat#sc-393002; RRID: AB_2893006
Rabbit-KRT6a	LS Bio	Cat#LS-B12036
Rabbit-KRT8	LS Bio	Cat#LS-B7928
Rabbit-KRT4	ThermoFisher	Cat#MA5-37810; RRID: AB_2897734
Rabbit-KRT5	Abcam	Cat#ab52635; RRID: AB_869890
Mouse-aSMA-FITC	Millipore Sigma	Cat#F3777; RRID: AB_476977
Mouse, anti-P63	Biomedical	Cat#CM-163-A; RRID: AB_10582730
Rabbit-MUC5B	Cloud-Clone Corp	Cat#PAA684Mu01
Rabbit-SNAP25	Sigma-Aldrich	Cat#S9684; RRID: AB_261576
Goat-GNAT3 (a-gustducin)	Aviva Systems Biology	Cat#OEAB00418
Mouse-TUJ1	Novus	Cat#BAM1195; RRID: AB_456859
Rabbit-AGR2	Cell Signaling Technology	Cat#13062S
Rabbit-Dclk1	Abcam	Cat#ab37991
Mouse-RFP	Abcam	Cat#ab125244; RRID: AB_10973556
Mouse-FOXJ1	eBioscience/Fisher	Cat#5013931
Mouse-Acetylated tubulin	Sigma	Cat#T7451; RRID: AB_609894
Rabbit-SCGB1A1	Abcam	Cat#ab213203; RRID: AB_2650558
Rat- SCGB3A2	R&D Systems	Cat#381707
Goat anti-Rabbit IgG Alexa Flour 488	ThermoFisher	Cat#A27034; RRID: AB_2536097
Goat anti-mouse IgG, Cy3^TM^	Jackson Immuno Research	Cat#115-166-003; RRID: AB_2338699
Goat anti-rat IgG, Cy3^TM^	Jackson Immuno Research	Cat#112-165-003; RRID: AB_2338240
BV510-conjugated anti-CD45	BioLegend	Cat#103138; RRID: AB_2563061
APC-FITC-conjugated anti-Epcam	BioLegend	Cat#118214; RRID: AB_1134102
PE-conjugated anti-CD31	BioLegend	Cat#102508; RRID: AB_312915
CD11b: Brilliant Violet 450	BioLegend	Cat#75-0112-U025; RRID: AB_2621936
Mm-Tmprss11a	Advanced Cell Diagnostics	Cat#1232761
Mm-Nkx2.1	Advanced Cell Diagnostics	Cat#434721
Mm-Nkx3.1	Advanced Cell Diagnostics	Cat#472111
Mm-Kcnj16	Advanced Cell Diagnostics	Cat#492481
Mm-Il1a	Advanced Cell Diagnostics	Cat#440398
Mm-Dmbt1	Advanced Cell Diagnostics	Cat#418561
Mm-Igfbp2	Advanced Cell Diagnostics	Cat#405951
Mm-Lpo	Advanced Cell Diagnostics	Cat#403521
Mm-Slc34a2	Advanced Cell Diagnostics	Cat#449311

**Chemicals, peptides, and recombinant proteins**		

Tamoxifen	Sigma-Aldrich	Cat#T-5648-1G
RPMI 1640 Medium	Thermo Scientific	Cat#11875093
1 mM HEPES	Life Technology	Cat#15630080
Dispase	Roche	Cat#4942086001
DNase I	Roche	Cat#11284932001
1 mM CaCl2	Sigma-Aldrich	Cat#21115
1 mM MgCl2	Sigma-Aldrich	Cat#20303M
Collagenase D	Roche	Cat#11088858001
RBC Lysis Buffer	BioLegend	Cat#420301

**Critical commercial assays**		

RNAscope® Multiplex Fluorescent Reagent Kit V2	Advanced Cell Diagnostics	Cat#323100

**Deposited data**		

Single cell RNA-seq data for mouse larynx/trachea	Gene Expression Omnibus	GSE287495

**Experimental models: Organisms/strains**		

Mouse: Wildtype C57BL/6J	Jackson laboratory	JAX: 000664
Mouse: Chat-cre	Jackson laboratory	JAX: 031661
Mouse: Rosa-lxl-tdTomato (Ai14)	Jackson laboratory	JAX: 007914
Mouse: Ascl1-creERT2	Jackson laboratory	JAX: 012882

**Software and algorithms**		

RStudio (v2024.12.1 + 563)	RStudio Team	http://www.rstudio.com/
R (v4.5.0)	R Core Team	https://www.R-project.org/
Cell Ranger (v3.0.2)	10x Genomics	https://support.10xgenomics.com/single-cell-gene-expression/software/pipelines/latest/what-is-cell-ranger

### Experimental model and study participant details

All experimental procedures were performed in the American Association for Accreditation of Laboratory Animal Care (AAALAC)-certified laboratory animal facility at the University of California, San Diego (UCSD). All animal husbandry and experiments were conducted under approved Institutional Animal Care and Use Committee (IACUC) guidelines. Wild-type C57BL/6 (JAX 000664)*, Ascl1*^*CreERT2*^ (JAX 012882), *Rosa*^*lxl-tdTomato*^ (*Ai14*, JAX 007914), and *Chat*^*cre*^ (JAX 031661) lines were purchased from the Jackson lab. All the *cre* lines we used in this study were kept in C57BL/6 background. All *cre* driver lines in heterozygous form are viable and fertile, and no abnormal phenotypes were detected. Both male and female mice were used in the experiment. Adult mice were 8–10 weeks of age for all experiments.

### Method details

#### Tissue collection and immunofluorescence staining

Mice were euthanized by CO2 inhalation followed by transcardial perfusion with PBS to remove circulating blood. The larynx-trachea was isolated and fixed overnight in 1%PFA or immediately embedded in OCT, flash frozen using 2-Methylbutane and liquid nitrogen and stored at −80°C. Using a cryostat, the larynx-trachea was then sectioned (12 μm) and stored at −20°C. Unfixed tissue was then washed in PBS for 5 min to remove OCT, heated to boiling in 10 mM citrate buffer (pH 9) for antigen retrieval and treated with 0.5% Triton X-100 in PBS for 15min. All sections were processed for immunostaining following a standard protocol.^27^ *Ascl1* recombination was induced via Tamoxifen IP administration with dose of 100 mg/kg for three consecutive days. All primary and secondary antibodies used are listed in Table S6. Primary antibodies were applied overnight at 4°C, while secondary antibodies were applied for 1h at room temperature. Sections were incubated with DAPI (1:1,000 ratio) for 10 min at RT. Slides were mounted and coverslipped with Prolong Diamond mounting media (Fisher P36970), cured flat at room temperature in the dark for 24 h, and stored at 4°C. Each experiment was replicated at least twice for all timepoints and targets assessed.

#### RNAscope *in situ* hybridization

All staining procedures were performed using the RNAscope Fluorescent Multiplex Kit V2 (Advanced Cell Diagnostics, no. 323100) following the manufacturer’s instructions. The following probes from Advanced Cell Diagnostics were used: Mm-*Tmprss11a* (no. 1232761), Mm-*Nkx2.1* (no. 434721), Mm-*Nkx3.1* (no. 472111), Mm-*Dmbt1* (no. 418561), Mm-*Igfbp2* (no. 405951), Mm-*Lpo* (no. 403521), Mm-*Slc34a2* (no. 449311), Mm-*Il1a* (no. 440398), Mm-*Kcnj16* (no. 492481).

#### Tissue processing and flow cytometry

Tissue spanning from the lower pharynx and larynx, beginning at the base of the tongue and tip of the epiglottis and extending distally to the tracheobronchial carina (larynx-trachea), was mechanically dissociated by finely mincing with razor blades in solution containing 5 mL of RPMI 1640 (Thermo Scientific) with 10% fetal bovine serum, 1 mM HEPES (Life Technology), 1 mM MgCl_2_ (Life Technology), 1 mM CaCl_2_ (Sigma), 0.5 mg mL^−1^ collagenase D and type I/dispase (Roche), and 0.25 mg DNase I (Roche). Minced tissue was then digested by shaking at around 150 rpm for 30 min at 37°C. Following incubation, upper airway pieces were mechanically dissociated further by straining through a MACS 70 μm filter. Red blood cells were removed by the addition of 1 mL of RBC lysis buffer (BioLegend) to each tube and incubation at room temperature for 1 min. Single-cell suspensions were pelleted (1,500 rpm, 4°C, 5 min), counted with a hemocytometer and diluted to around 1 × 10^6^ cells ml^−1^. Diluted cells were stained with Fc blocking antibody (5 mg mL^−1^, BD). To isolate epithelial lineage, cells were then incubated with the following specific surface marker antibodies: (Immune) 1:1,000 BV510-conjugated anti-CD45 (BioLegend, no. 103138), (Epithelial) 1:1,000 APC-FITC-conjugated anti-Epcam (BioLegend, no. 118214), and (Endothelial) 1:1,000 PE-conjugated anti-CD31 (BioLegend, no. 102508). Cells were then stained using live/dead dye (CD11b: Brilliant Violet 450, BioLegend, cat#75-0112-U025) before being resuspended in 2% FBS +1:2,000 DAPI. All FACS sorting was done on a BD FACSAria Fusion Sorter (BD Biosciences) analyzer with three lasers (405, 488 and 640 nm) at the Flow Cytometry Core at VA San Diego Health Care System and San Diego Veterans Medical Research Foundation. All data were further analyzed and plotted with FlowJo software (Tree Star).

### Quantification and statistical analysis

Data quality control, normalization, variable genes selection, principal component analysis (PCA), uniform manifold approximation and projection (UMAP), and differential gene expression testing were conducted using the Seurat (v4.0.5) pipeline.^64^^,^^65^

#### Preprocessing of 10X chromium scRNAseq data

Single cells were processed into complementary DNA libraries using Chromium Single Cell 3′ v3 kit (10X Genomics, Pleasanton, CA) and sequencing was carried out on the NovaSeq (Illumina) platform. The CellRanger software package from 10X Genomics (v3.0.2) was used to align raw reads onto the mouse reference genome (GRCm38) and generate single-cell gene barcode matrices. CellBender (v0.3.0)^66^ was then used to remove technical artifacts and ambient RNA to produce improved estimates of gene expression. To ensure the exclusion of potential thyroid epithelial contamination, we manually removed the thyroid gland during sample preparation and computationally filtered clusters expressing thyroid-specific genes (*Folr1, Tg, Foxe1*) from our dataset. Single cells below 15% mitochondrial reads and between 200 and 7,500 unique genes were considered high-quality cells and were filtered for further analyses. Additional filtering included a minimum UMI cutoff of 2 and minimum cell count cutoff of 10. LogNormalize global-scaling normalization method was applied with regressing variables cell cycle, S.Score, G2M.Score, and mitochondrial RNA level to remove confounding effect on downstream clustering. In addition, DoubletFinder (v2.0)^67^ was used to remove doublets and LogNormalize was again used to normalize feature expression.

#### Dimensionality reduction using PCA and UMAP

Selection of variable genes was implemented with the function *FindVariableFeatures* with method = “vst.” The top 3,000 highly variable genes were centered and scaled with function *ScaleData*. The first 50 PCs were calculated via function *RunPCA*, after which the function *RunUMAP* was applied to generate UMAP embeddings for 2-dimensional visualization.

#### Integration, clustering, and downstream analysis

CCA^68^ was used to integrate dataset across two experimental batches for a total of *n* = 8 mice (4 males, 4 females). In total 3,617 epithelial single cells were recovered for analysis. To determine dimensions for optimized clustering, we evaluated the optimal cutoff using an elbow plot and settled on using the first 50 principal components for clustering and projection with UMAP. Clustering resolutions was set to 0.8 (Seurat default), resulting in 19 interim clusters representing potential unique cell populations. Following manual inspection of top markers, we combined several clusters with shared markers to ensure that annotated clusters would show unique transcriptional profiles, resulting in 18 distinct clusters. For rigorous definition of marker genes in each cluster, we performed unsupervised and supervised analysis to screen each cluster’s top marker genes (*FindAllMarkers*) using ViolinPlot, FeaturePlot and DotPlot. To complement the default *FindAllMarkers* “Wilcoxon rank-sum test,” we utilized a “Model-based analysis of single-cell transcriptomics (MAST) test” that is particularly well-suited for single-cell data because it uses a hurdle model to account for both the dropout rate (the probability that a gene is not detected in a cell) and the continuous expression level among cells that do express the gene. Cell clusters were referenced against available literature.^8^^,^^13^^,^^16^ Cell clusters were validated with RNAscope and immunofluorescence staining (see main figures). We present a list of the top 200 differentially expressed marker genes for all cell clusters, analyzed using both Wilcoxon and MAST tests, in Table S3. Using FDR-corrected Fisher’s combined *p*-value <0.001 and a minimum avg_log2FC of 2.0, we identified the top 100 genes per cluster, listed in Table S1. High-throughput sequence data is available at GEO. Once exclusively differentially expressed genes were identified, we performed tests of enrichment using Gene Ontology (GO) annotations utilizing Enrichr (v2024)^69^ as previously described.^70^

## Supplemental information

Document S1. Figures S1–S6

Table S1. Consensus marker genes for cell-type clusters from 3′ droplet-based scRNA-seq data (3,617 cells)

Table S2. Cell-type enriched TFs, GPCRs and KRTs from 3′ droplet-based scRNA-seq data (3,617 cells)

Table S3. Differentially expressed genes (top n = 200) across all cells identified using Seurat’s *FindAllMarkers* with both Wilcoxon rank-sum and MAST tests

Table S4. Table of significantly differentially expressed marker genes comparing basal-larynx versus basal-trachea versus basal-myoepithelial populations

Table S5. Table of significantly differentially expressed marker genes comparing club-proximal versus club-distal populations
