# Supplementary material for: Cellular heterogeneity and patterning strategies as revealed by upper respiratory epithelium single cell atlas
Source: iScience. 2025 Jun 7;28(7):112845. doi: 10.1016/j.isci.2025.112845 (PMC12221713; doi:10.1016/j.isci.2025.112845)
Supplement: Document S1. Figures S1–S6 [file mmc1.pdf]

iScience, Volume *28*

Supplemental information

Cellular heterogeneity and patterning strategies as revealed by upper respiratory epithelium single cell atlas

Alexander G. Foote and Xin Sun

Supplementary figures, tables, and legends

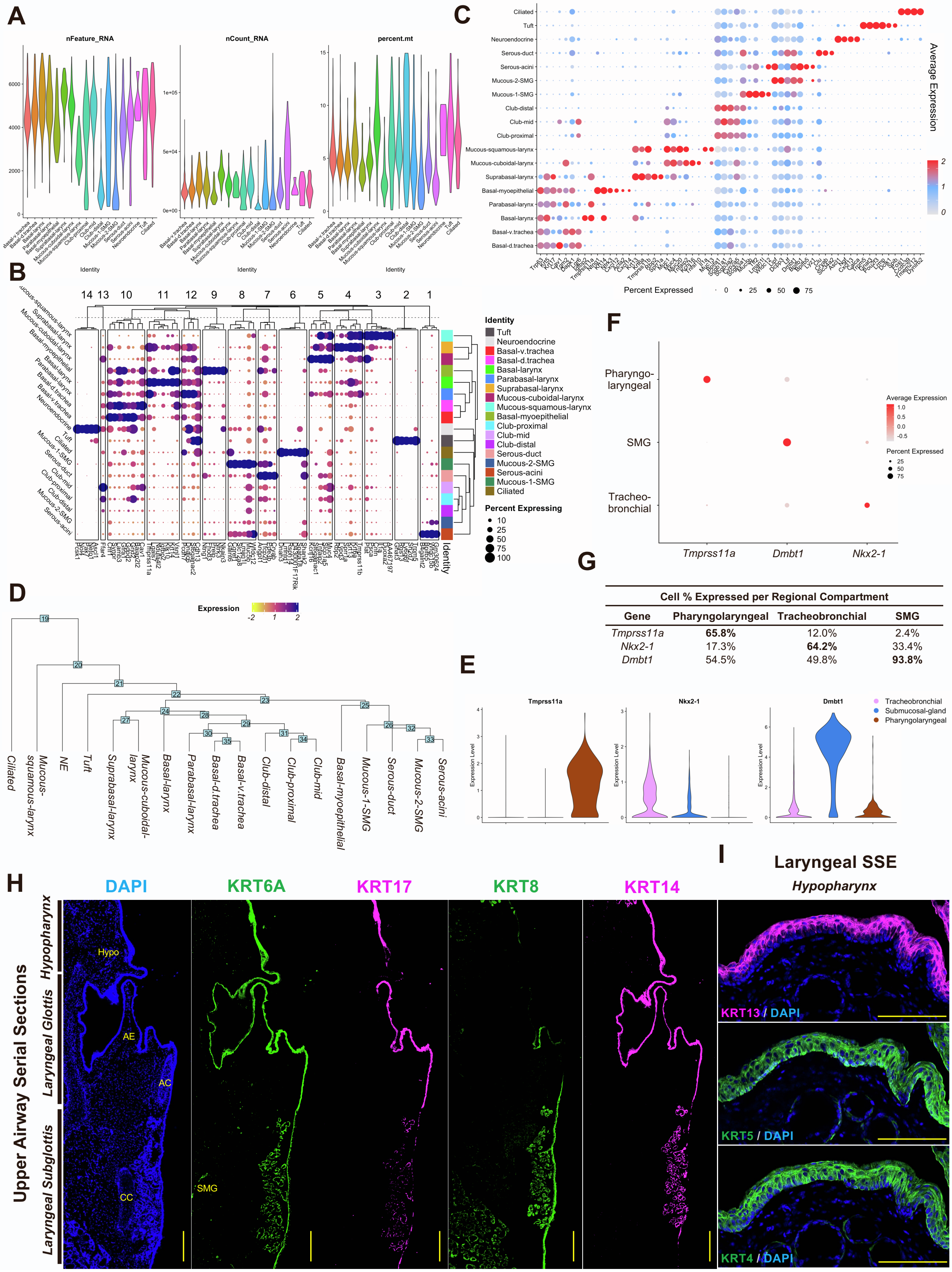

Cell % Expressed per Regional Compartment

Gene	Pharyngolaryngeal	Tracheobronchial	SMG
Tmprss11a	**65.8%**	12.0%	2.4%
Nkx2-1	17.3%	**64.2%**	33.4%
Dmbt1	54.5%	49.8%	**93.8%**

Figure S1. Comprehensive Analysis of Airway scRNAseq Dataset and Regional Marker Expression.

A. Quality control data including nFeature RNA (the number of genes detected in each cell), nCount RNA (the total number of molecules detected within a cell) and mitochondrial percentage of our integrated airway scRNAseq dataset.

B. Unsupervised clustering with Seurat default package for cell type annotation.

C. Supervised clustering exhibiting top known gene markers for cell type annotation.

D. Dendrogram displaying hierarchical relationships between clusters of cells using ‘PlotClusterTree’ Seurat package.

E. Top differentially expressed gene markers establishing macro-anatomical regional specificity.

F. Dot plot displaying both percent of cells expressing each marker and average expression level per regional compartment.

G. Calculation of the percentage of cells expressing unique transcript markers within each regional compartment.

H. Immunofluorescence of upper airway coronal serial sections in split-channel view indicating Keratins differential regional expression.

I. KRT4, KRT5 and KRT13 basal-to-luminal expression pattern in SSE of the hypopharynx. DAPI is in blue. Images are 20X magnification. Scale bar represents 100μm (I) and 500μm (H). AE aryepiglottis, AC arytenoid cartilage, CC cricoid cartilage, SMG submucosal gland.

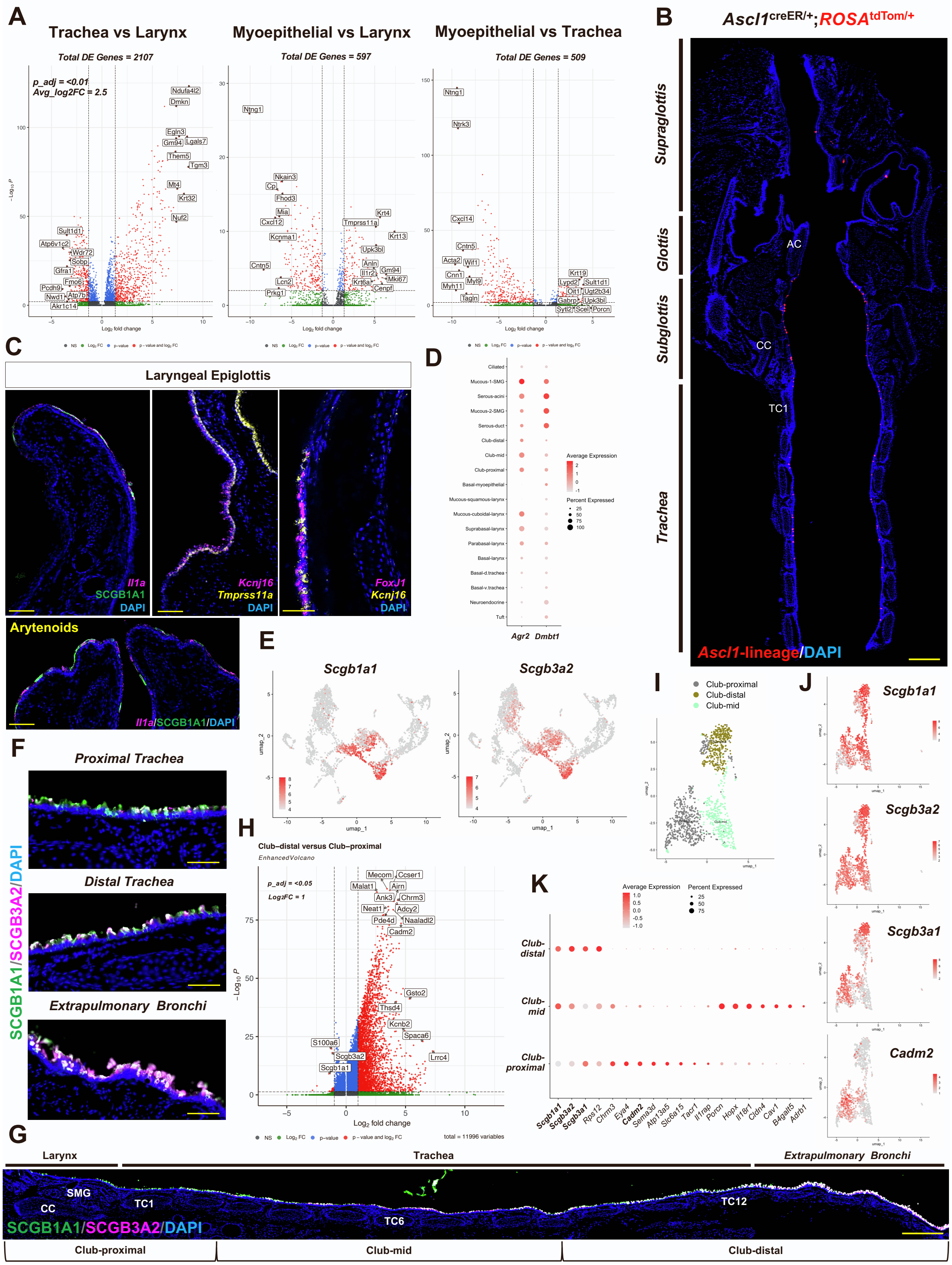

A
Trachea vs Larynx
Total DE Genes = 2107
p_adj = <0.01
Avg_log2FC = 2.5
Ndufa4l2
Dmkn
Egln3
Gm94
Lgals7
Them5
Tgm3
Mt4
Krt32
Nuf2
Sult1d1
Atp6v1c2
Wdr72
Sobp
Gfra1
Fmo6
Pcdh9
Atp7b
Nwd1
Akr1c14
Myoepithelial vs Larynx
Total DE Genes = 597
Ntng1
Nkain3
Cp
Fhod3
Mia
Cxcl12
Kcnma1
Cntn5
Lcn2
Prkg1
Krt4
Tmprss11a
Krt13
Upk3bl
Anln
Il1r2
Gm94
Krt6a
Mki67
Cenpf
Myoepithelial vs Trachea
Total DE Genes = 509
Ntng1
Ntrk3
Cxcl14
Cntn5
Acta2
Wif1
Cnn1
Myl9
Myh11
Tagln
Krt19
Lypd2
Sult1d1
Oit1
Ugt2b34
Gabrp
Upk3bl
Sytl2
Scel
Porcn
Log2 fold change
NS
Log2 FC
p-value
p-value and log2 FC
B
Ascl1creER/+;ROSAtdTom/+
Supraglottis
Glottis
Subglottis
Trachea
AC
CC
TC1
Ascl1-lineage/DAPI
C
Laryngeal Epiglottis
Il1a
SCGB1A1
DAPI
Kcnj16
Tmprss11a
DAPI
FoxJ1
Kcnj16
DAPI
Arytenoids
Il1a/SCGB1A1/DAPI
D
Ciliated
Mucous-1-SMG
Serous-acini
Mucous-2-SMG
Serous-duct
Club-distal
Club-mid
Club-proximal
Basal-myoepithelial
Mucous-squamous-larynx
Mucous-cuboidal-larynx
Suprabasal-larynx
Parabasal-larynx
Basal-larynx
Basal-d.trachea
Basal-v.trachea
Neuroendocrine
Tuft
Agr2
Dmbt1
Average Expression
Percent Expressed
E
Scgb1a1
Scgb3a2
umap_1
umap_2
F
Proximal Trachea
Distal Trachea
Extrapulmonary Bronchi
SCGB1A1/SCGB3A2/DAPI
G
Larynx
Trachea
Extrapulmonary Bronchi
SMG
CC
TC1
TC6
TC12
SCGB1A1/SCGB3A2/DAPI
Club-proximal
Club-mid
Club-distal
H
Club-distal versus Club-proximal
EnhancedVolcano
p_adj = <0.05
Log2FC = 1
Mecom
Ccser1
Malat1
Airn
Ank3
Chrm3
Neat1
Adcy2
Pde4d
Naaladl2
Cadm2
Gsto2
Thsd4
Kcnb2
Spaca6
Lrrc4
S100a6
Scgb3a2
Scgb1a1
Log2 fold change
total = 11996 variables
I
Club-proximal
Club-distal
Club-mid
J
Scgb1a1
Scgb3a2
Scgb3a1
Cadm2
K
Average Expression
Percent Expressed
Club-distal
Club-mid
Club-proximal
Scgb1a1
Scgb3a2
Scgb3a1
Rps12
Chrm3
Eya4
Cadm2
Sema3d
Atp13a5
Slc6a15
Tacr1
Il1rap
Porcn
Hopx
Il18r1
Cldn4
Cav1
B4galt5
Adrb1

Figure S2. Differential Gene Expression and Regional Profiling of Airway Cell Subsets.

A. Volcano plot exhibiting differential gene (DE) expression of basal cell populations.

B. 20X coronal immunofluorescent section from Ascl1$^{creERi/+}$;ROSA$^{tdTom/+}$ reporter murine tissue exhibiting *Ascl1*+lineage marked neuroendocrine cells (red) of the upper airway.

C. Mucous-producing cells in pharyngolaryngeal epithelium exhibiting *Il1a* or *Kcnj16* expression.

D. Dot plot displaying *Agr2* enrichment in Mucous-1-SMG cells, alongside *Dmbt1* expression.

E. Feature plot exhibiting cell specific expression of *Scgb1a1* and *Scgb3a2* transcripts.

F. Immunofluorescence exhibiting increased abundance and expression of SCGB1A1+ and SCGB3A2+ proteins in distal and extrapulmonary bronchi.

G. 20X coronal immunofluorescent section exhibiting *Scgb1a1* and *Scgb3a2* expression along the proximal-to-distal airway axis

H. Pairwise DE analysis of club-proximal versus club-distal exhibiting increased *Scgb1a1* and *Scgb3a2* expression to distal airway club cells.

I. Integrated UMAP of club cell subset.

J. Feature plot exhibiting cell specific expression *Scgb1a1*, *Scgb3a2, Scgb3a1,* and *Cadm2* transcripts from our club cell subsetted scRNAseq dataset.

K. DotPlot showing top DE genes associated with functional enrichment analysis. DAPI is in blue. Images are 20X magnification. Scale bar represents 100μm (C,F) and 500μm (B,G). AC arytenoid cartilage, CC cricoid cartilage, TC tracheal cartilage, SMG submucosal gland.

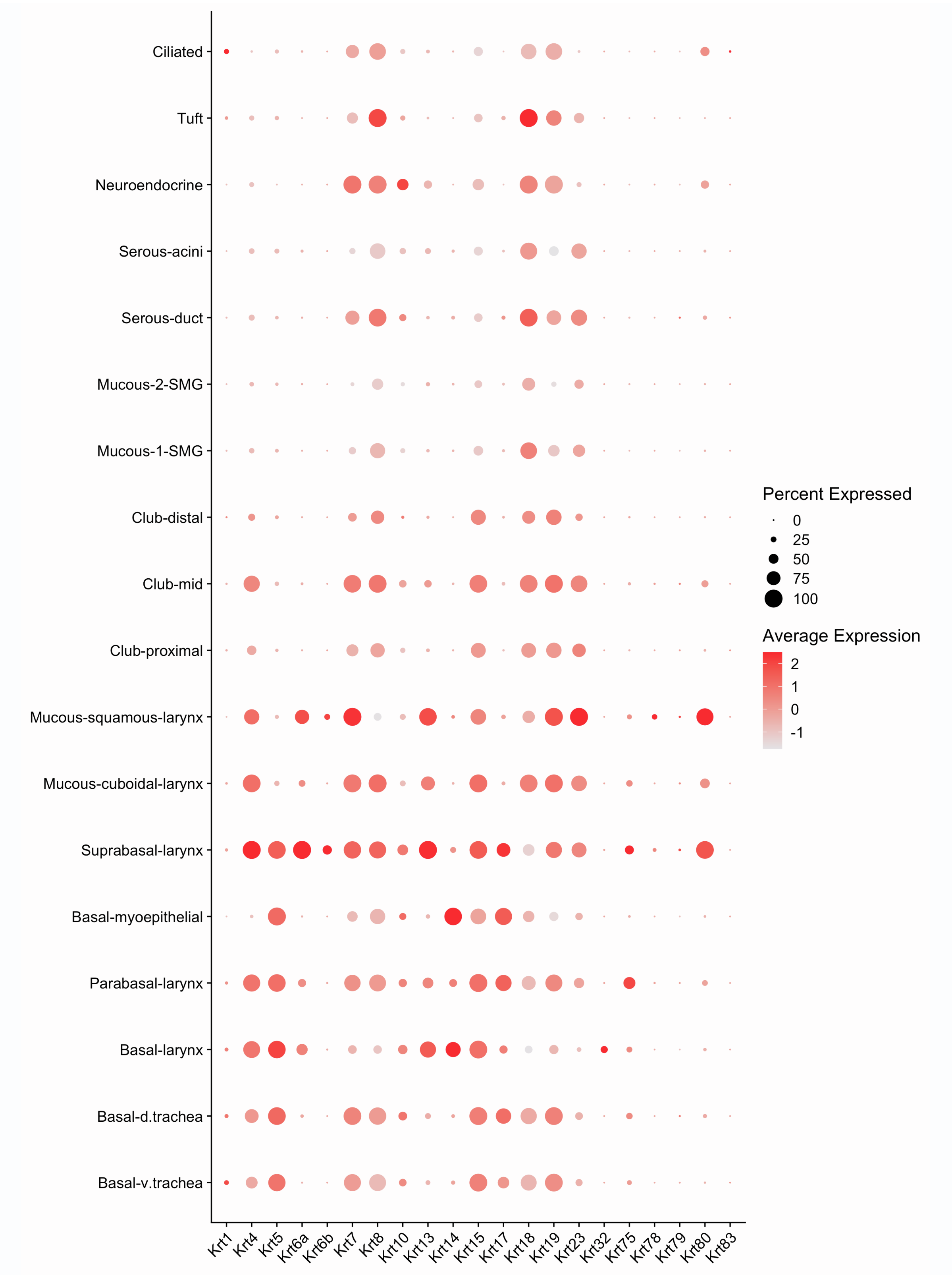

Figure S3. DotPlot exhibiting comprehensive profiling of Keratin diversity across all epithelial cell types in our integrated scRNAseq dataset.

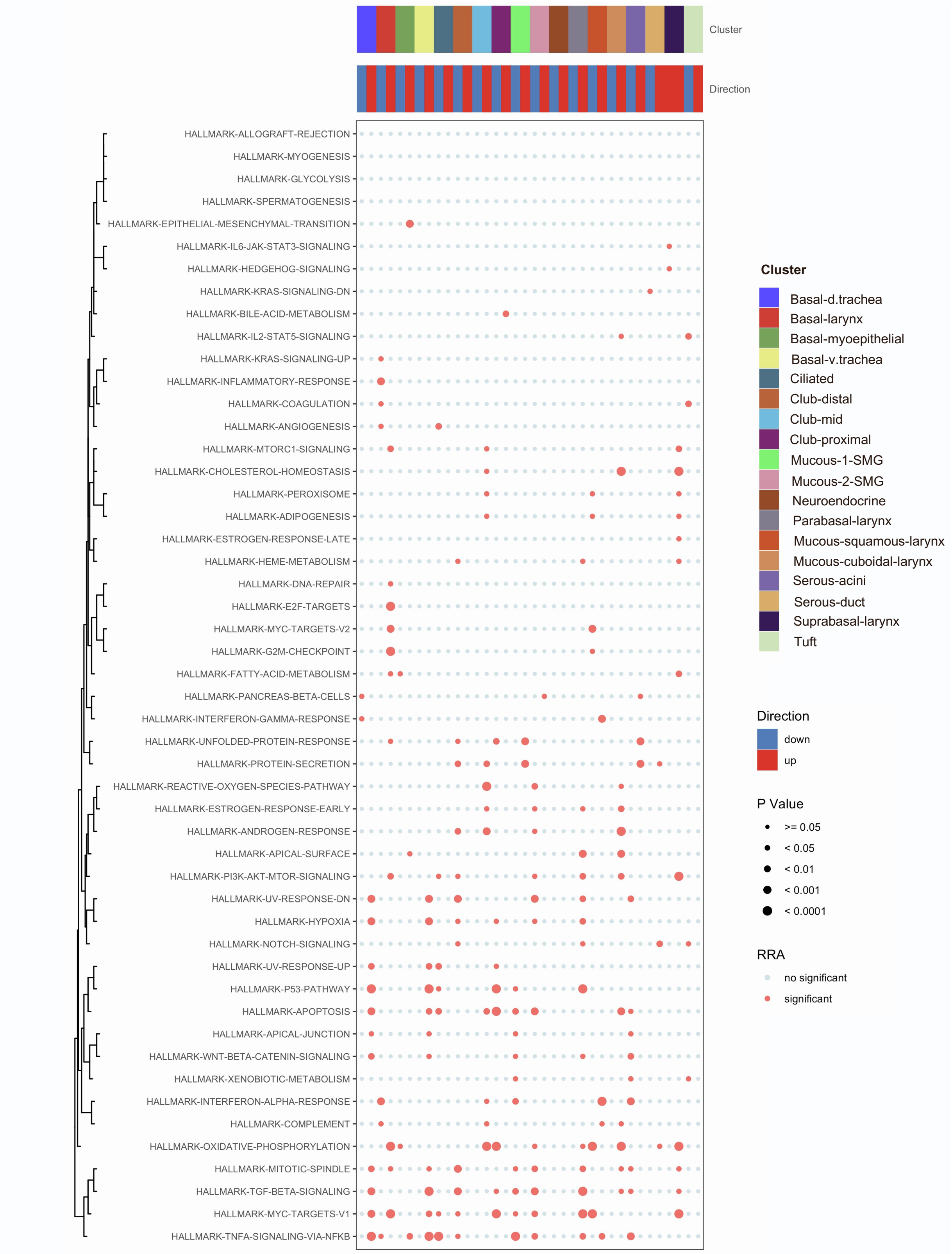

Figure S4. irGSEA hallmark pathways analysis of differentially expressed (DE) genes across all epithelial cell types in our integrated scRNAseq dataset.

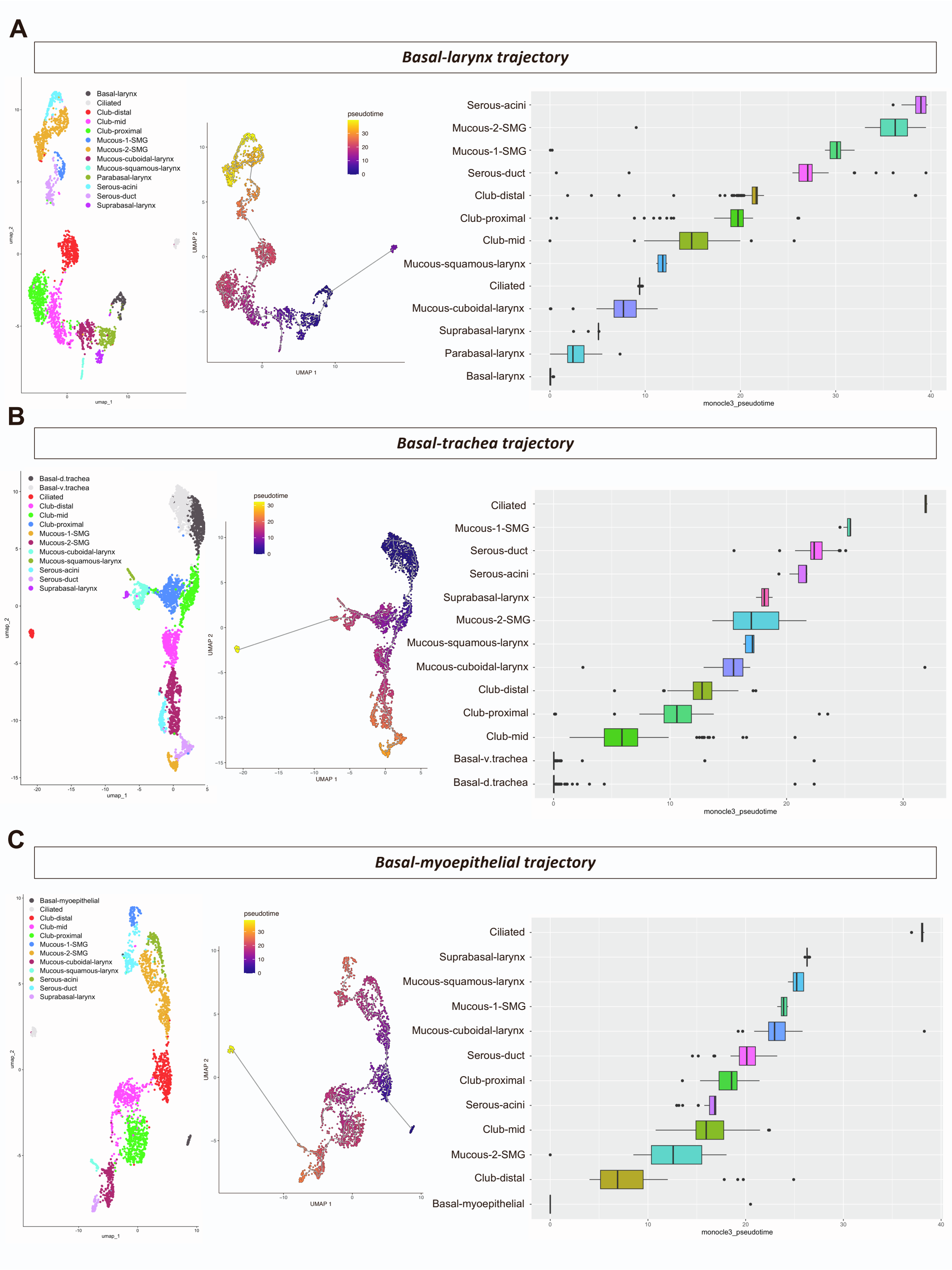

Figure S5. Trajectory Mapping of Upper Airway Basal Cell Subsets Using Monocle 3. Subset UMAP plots of integrated scRNAseq dataset from upper airway, analyzed with Monocle 3 trajectory mapping and pseudotime boxplots, displaying (A) basal-larynx, (B) basal-trachea, and (C) basal-myoepithelial cell origins.

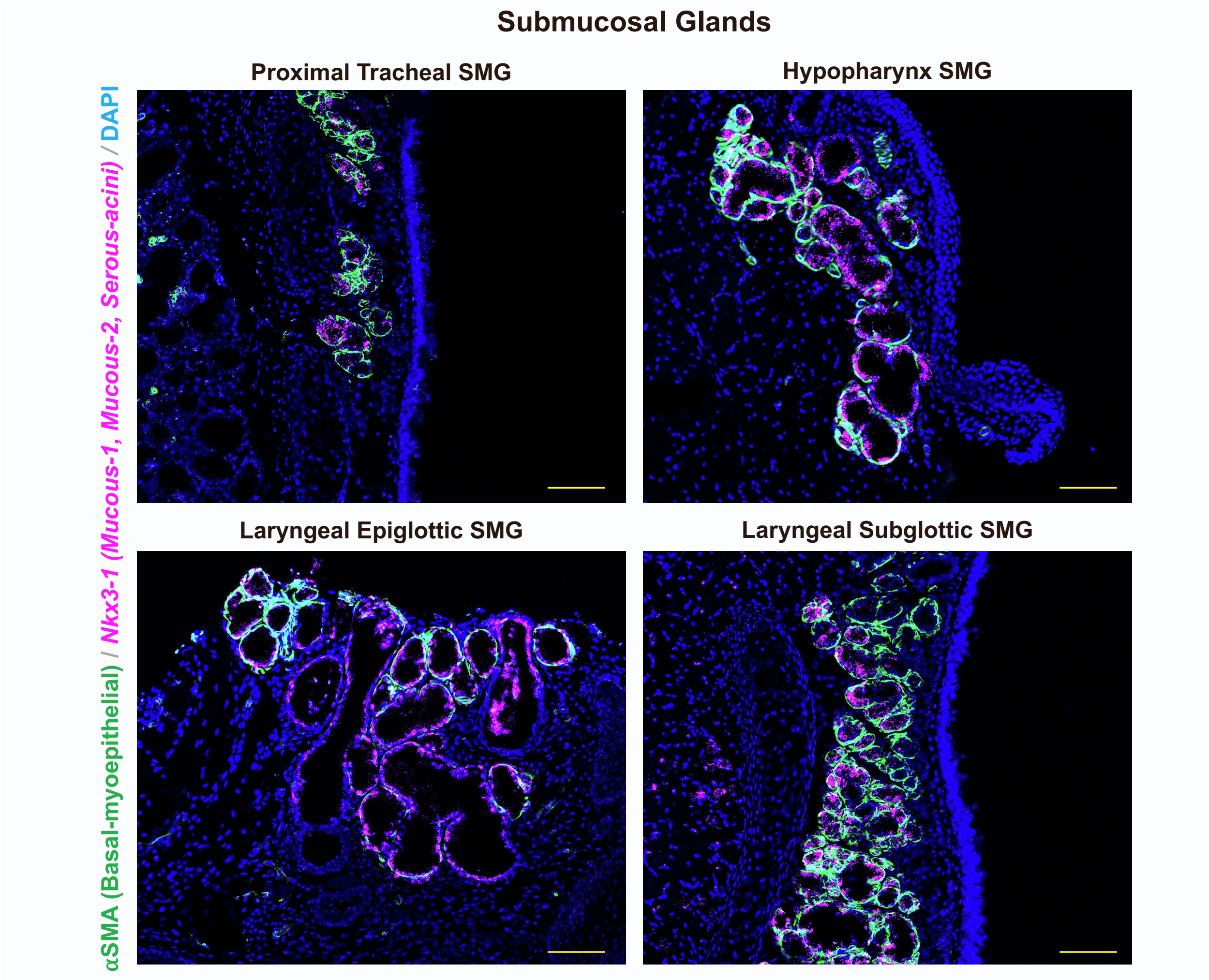

Figure S6. Conserved Protein Expression Patterns in Glandular Regions via Immunostaining.

Additional immunostaining for *Nkx3-1* (Mucous-1, Mucous-2, Serous-duct) and αSMA (Basal-myoepithelial) demonstrating conserved protein expression patterns across all glandular regions. Images are 20X magnification. Scale bar represents 100µm. SMG submucosal gland.
